# Management of Primary Spontaneous Pneumothorax in the Emergency Department: A Cost-Effectiveness Analysis

**DOI:** 10.1016/j.acepjo.2025.100209

**Published:** 2025-07-02

**Authors:** Margaret L. Davis, Jane Hall, R. Andrew Taylor, Robert Klemisch, Farhood Farjah, M. Kennedy Hall

**Affiliations:** 1 Department of Emergency Medicine, University of Washington, Seattle, Washington, USA; 2 Department of Emergency Medicine, Yale University School of Medicine, New Haven, Connecticut, USA; 3 Department of Biomedical Informatics and Data Science, Yale University School of Medicine, New Haven, Connecticut, USA; 4 Department of Surgery, University of Washington, Seattle, Washington, USA

**Keywords:** primary spontaneous pneumothorax, cost-effectiveness analysis, health economics, net monetary benefit, observation, needle aspiration, small-bore chest tube, decision-analytic modeling, probabilistic sensitivity analysis

## Abstract

**Objectives:**

Primary spontaneous pneumothorax (PSP) is a common emergency condition among healthy individuals that presents significant management challenges, particularly in cases of moderate-to-large collapse, which have traditionally required intervention. Recent trials, including a landmark-randomized trial, have demonstrated effectiveness in 4 separate strategies: (1) observation only, (2) needle aspiration, (3) small-bore chest tubes with a 1-way valve, and (4) small-bore chest tubes with continuous suction. We compare the cost-effectiveness of these 4 strategies.

**Methods:**

We conducted a cost-effectiveness analysis from a US healthcare system perspective to compare 4 management strategies for moderate-to-large PSP in stable adult emergency department patients. Using published randomized controlled trials and secondary data, we developed a decision-analytic model with a lifetime horizon. Costs (2022 USD), quality-adjusted life-years, and net monetary benefit (NMB) were calculated at a $50,000/quality-adjusted life-years willingness-to-pay threshold and 3% annual discount. We performed 1-way and probabilistic sensitivity analyses to test the robustness of findings.

**Results:**

Base case analysis identified observation only as the most cost-effective strategy, achieving the highest NMB ($43,696). The NMB values for small-bore chest tubes with a 1-way valve, needle aspiration, and small-bore chest tube with continuous suction were $33,520, $29,465, and $12,831, respectively. Sensitivity analyses indicated that factors such as initial success rates for observation and needle aspiration influenced model outcomes, whereas probabilistic simulations confirmed observation only as the dominant strategy.

**Conclusions:**

Observation alone is the most cost-effective initial strategy for stable moderate-to-large PSP, although its feasibility may be limited. Among interventional options, a small-bore chest tube with a 1-way valve offers the best value and enables outpatient management. Further studies in diverse settings are needed to confirm the practicality of observation only care and consider individual patient factors.

**The Bottom Line**

Observation-only is the most cost-effective initial strategy for managing stable, moderate-to-large primary spontaneous pneumothorax (PSP), achieving the highest net monetary benefit modeling United States Health System costs. Among interventions, a small-bore chest tube with a one-way valve is the most cost effective and allows for outpatient management. Needle aspiration and chest tube to suction were less cost-effective. While observation is dominant in cost-effectiveness, its feasibility in U.S. emergency departments may be limited. Further research is needed to assess its practicality and guide patient-centered management decisions.

## 1 Introduction

### 1.1 Background

Primary spontaneous pneumothorax (PSP) is a relatively common medical condition characterized by the sudden accumulation of air within the pleural cavity without an external cause that frequently affects young, otherwise healthy individuals with an incidence of 1.2-18 cases per 100,000 population.^1^^,^^2^ Large PSP is defined by radiological criteria, usually involving the presence of a pneumothorax occupying more than one-third of the hemithorax or any pneumothorax associated with clinical signs of respiratory distress.^3^ Smaller pneumothoraces often resolve spontaneously or with minimal intervention,^4^ but the management of large PSP presents unique challenges due to its potential for significant respiratory compromise and increased risk of tension pneumothorax. As such, large PSP have traditionally been managed with an intervention. Traditional treatment involves inserting a small-bore chest tube that is connected to continuous suction to evacuate the pneumothorax. Increasingly, other strategies are used to manage PSPs.^5^ These include simple needle aspiration and placement of a small-bore chest tube with a 1-way valve. Needle aspiration for PSP is performed by placing a needle with a catheter into the pleural cavity and evacuating the air using a syringe, followed by removal of the catheter. Alternatively, a small-bore chest tube with 1-way valve is inserted to allow air to escape the pleural cavity and not re-enter; hence, the tube does not need to be connected to continuous suction, enabling outpatient management. Most recently, a landmark-randomized trial showed that for stable, moderate-to-large PSPs, an observation only approach was noninferior to interventional treatment in achieving radiographic resolution at 8 weeks.^6^ Even patients with large pneumothoraces, including 1 with radiographic—but no physiologic—signs of tension, were discharged safely, demonstrating the viability of a conservative management strategy.

### 1.2 Importance

As health care systems face mounting pressure to improve the quality of care while controlling costs, determining the most cost-effective approach to managing large PSPs has broad implications. For patients, less-invasive, conservative strategies may reduce procedure-related discomfort, shorten emergency department (ED) stays, and expedite the return to normal activities. From an operational standpoint, adopting more efficient methods can decrease hospital resource use, minimize physician time spent on invasive interventions, and improve patient throughput. Conservative strategies including observation are already used outside of the United States and are incorporated into the British Thoracic Society guidelines.^3^ Ultimately, identifying cost-effective treatments enables decision-makers to optimize both clinical outcomes and resource allocation, ensuring patients receive high-value, patient-centered care.

### 1.3 Goals of This Investigation

The aim of this study was to identify the most cost-effective strategy among the following 4 treatment options: (1) observation only, (2) needle aspiration, (3) small-bore chest tube with 1-way valve, and (4) small-bore chest tube to suction in terms of cost-effectiveness for ED management of a large PSP.

## 2 Methods

### 2.1 Study Design

We performed a cost-effectiveness analysis of 4 treatment strategies for PSP by using TreeAge Pro 2022 (TreeAge Software Inc) following published guidelines and the consolidated health economic evaluation reporting standards.^7^ Treatment strategies were compared using net monetary benefit (NMB) to help clinicians identify the option with the highest value for both patient outcomes and financial efficiency.^8^^,^^9^ This study was based on a literature review and exempt from review by our institutional review board.

### 2.2 Population and Treatment Strategies

**Figure 1:**
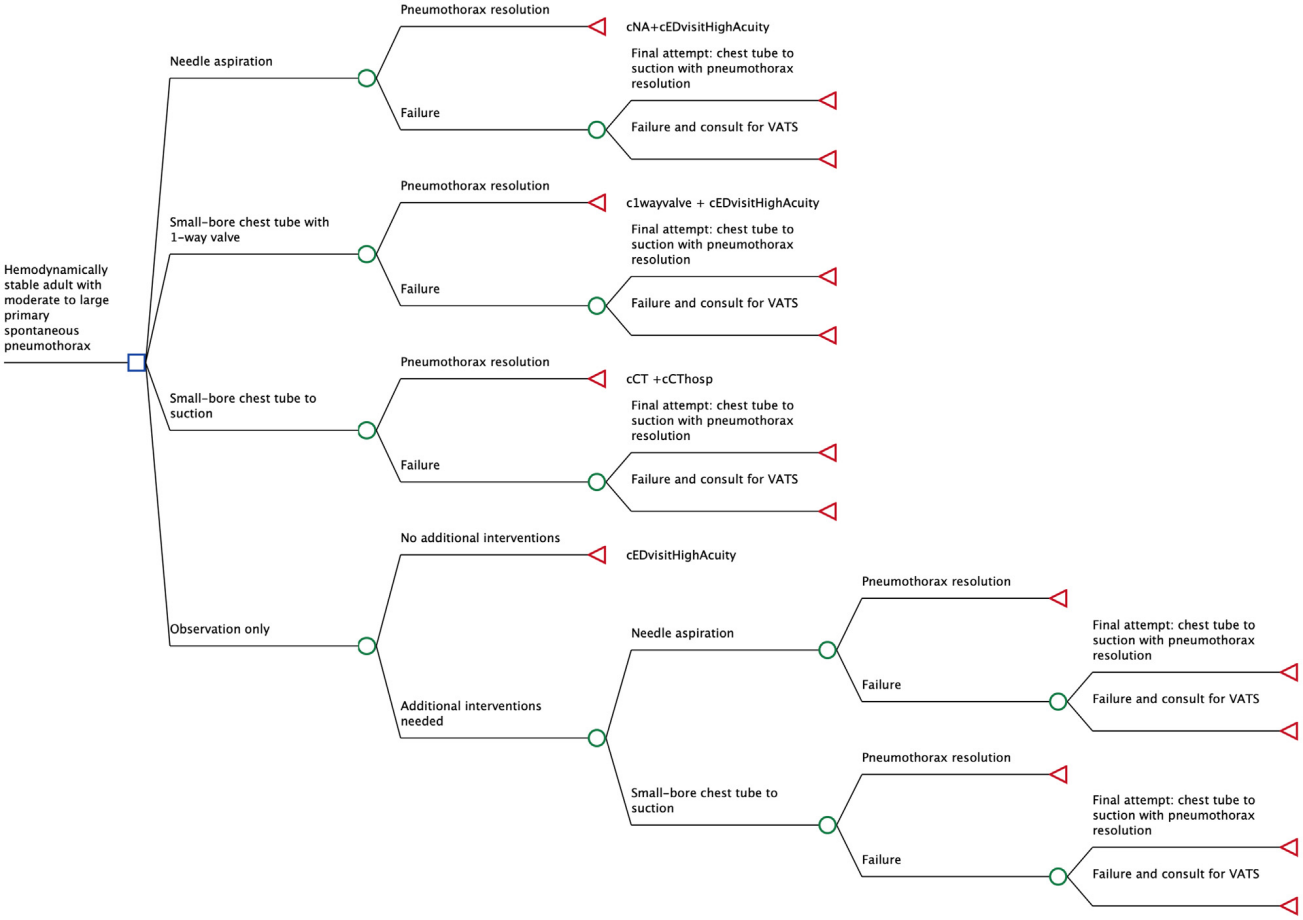
Decision analysis tree. The model evaluates the difference between costs and outcomes in relation to the selected treatment strategy for managing a moderate-to-large primary spontaneous pneumothorax. The decision node (square) signifies the selection of a management approach, whereas chance nodes (circles) represent probabilistic events with multiple potential outcomes beyond the decision maker’s control. Terminal nodes (triangles) indicate the conclusion of each scenario.

For adult patients in the ED with moderate-to-large PSP, we examined the following 4 treatment strategies described above in terms of increasing invasiveness: (1) observation only, (2) needle aspiration, (3) small-bore chest tube with 1-way valve, and (4) small-bore chest tube to suction. All patients initially received a chest radiograph, supplemental oxygen, and a high-complexity ED evaluation. In the needle aspiration pathway, failure to resolve the pneumothorax led to the placement of a small-bore chest tube on suction. For both needle aspiration and 1-way valve strategies, successful resolution prompted patient discharge. In the observation only strategy, patients were discharged if no procedures were required. All patients treated initially with small-bore chest tube suction were admitted. If suction failed to resolve the pneumothorax in any pathway, patients proceeded to consultation with a thoracic surgeon for consideration of video-assisted thoracoscopic surgery (VATS) bleb resection and mechanical pleurodesis. The model structure is shown in Figure 1.

### 2.3 Clinical Data

Model inputs used data from published randomized controlled trials (RCTs) (Table 1).^6^^,^^10^, ^11^, ^12^, ^13^, ^14^, ^15^, ^16^, ^17^, ^18^, ^19^, ^20^, ^21^, ^22^, ^23^, ^24^, ^25^, ^26^ The base case patient was a previously healthy 25-year-old adult with a normal life expectancy of 78 years based on the average age of onset of PSP.^10^ Rates of resolution of pneumothorax using each of the 4 treatment strategies were based on pooled data from the trials.

**Table 1:** Input probabilities.

Parameter	Base case analysis	Range for sensitivity analysis
Probability of initial success of observation only^6^^a^	84.6%	76.9%-89.6%
Probability of initial success of needle aspiration^11^, ^12^, ^13^, ^14^, ^15^, ^16^, ^17^, ^18^, ^19^, ^20^, ^21^, ^22^	65.7%	63.5%-95.2%
Probability of initial success of small-bore chest tube with 1-way valve^12^^,^^21^^,^^23^^,^^24^	76.9%	53%-91%
Probably of initial success of small-bore chest tube to suction^11^^,^^13^, ^14^, ^15^, ^16^, ^17^, ^18^, ^19^, ^20^, ^21^, ^22^, ^23^, ^24^	75.6%	63%-99%
Discount rate^25^	3%	1%-5%
Age (y)^26^	25	15-35
Life expectancy (y)^10^	78	60-82

a Reported as not needing additional intervention at index visit.

We conducted a standardized search in accordance with the Population, Intervention, Comparison, Outcome framework to answer the question “In adults presenting to the ED with moderate-to-large PSP, how does observation, needle aspiration, and chest tube with 1-way valve compared to chest tube to suction affect initial treatment success?” Our search included databases, such as Google Scholar and PubMed, and primary articles found in Cochrane Reviews in August 2022 using the terms “primary spontaneous pneumothorax,” “needle aspiration,” “manual aspiration,” “simple aspiration,” “percutaneous aspiration,” “one-way valve,” and “Heimlich valve.” The initial literature search yielded 1172 studies, of which the vast majority were excluded after a rigorous screening process due to methodological limitations, lack of relevance, or insufficient data. To ensure the analysis was based on robust and reliable evidence, studies were assessed against predefined inclusion criteria emphasizing methodological rigor and adherence to established standards for RCTs.^27^ Ultimately, 15 high-quality RCTs were identified and included, providing sufficient rigor and relevance to inform the analysis and enhance the validity of the findings (Table S1). Notably, only 1 randomized trial included an observation only strategy.^6^ Age-specific mortality for the general population was taken from the US life expectancy tables.^28^

### 2.4 Cost

Cost data were obtained from national averages in the United States using published costs from the Centers for Medicare and Medicaid Services.^29^, ^30^, ^31^ ED costs were estimated using professional and facility fees for each current procedural terminology code, and hospitalization costs were estimated using diagnostic related group codes (Table 2).^29^, ^30^, ^31^

**Table 2:** Cost estimates.

Description of service	CPT code	Median cost^a^, US $	Range for sensitivity analysis, US $
Emergency Department Visit-High Complexity^31^	99285	1097.43	877.94-1316.92
Chest Radiograph^29^	71045	94	75.20-112.80
Pleural Aspiration without imaging (needle aspiration)^29^	32554	683	546.40-819.60
Pleural Catheter w/o imaging (small-bore chest tube to suction or 1-way valve)^29^	32556	1931	1544.80-2317.20

Hospitalization costs	DRG code	Median cost, US $	Range for sensitivity analysis, US $

Admission for pneumothorax without complication^30^	201	28,858.84	$23,087.07-$34,630.61
Admission for VATS without complication^30^	165	86,135.69	69,908.55-103,362.83

CPT, current procedural terminology; DRG, diagnostic related group.

a Cost estimates include both physician and facility fees.

### 2.5 Utilities

Utilities represent the quality of life of an individual with a particular state of health numerically with 1 representing perfect health and 0 representing death. The utilities in our study were estimated based on published studies that utilized the Health Utilities Index, a standardized measurement of health utility.^32^ The long-term possible states of health considered in this study included the utility of a resolved PSP^33^ and the utility associated with a major complication resulting from a PSP or from subsequent procedures used to treat it.^34^ The long-term utility after an invasive lung procedure was estimated using the expertise of our thoracic surgery coauthor who used a published long-term disutility of moderate asthma as a surrogate for intermittent dyspnea.^34^ Utilities and the studies used to estimate them are listed in Table 3.^32^, ^33^, ^34^

**Table 3:** Utility estimates.

Utility	Base case analysis	Range for sensitivity analysis
Baseline state of health^32^	1	0.9-1
Primary spontaneous pneumothorax^33^	0.92	0.85-0.95
Invasive lung procedure^34^^a^	0.83	0.78-0.88

a Authors’ estimate.

### 2.6 Model Structure

Our model was structured starting with the decision of treatment strategy for a large PSP in a hemodynamically stable patient (Fig 1). The primary node represents the choice to pursue observation, needle aspiration, small-bore chest tube with 1-way valve, or small-bore chest tube to suction as the first treatment strategy. For the observation only strategy, success was defined as not needing an additional intervention at the index visit and safely discharged, despite that by definition at this time the pneumothorax was not resolved. For the other strategies, success was defined as the resolution of the pneumothorax. The model simulated events in the treatment course and concluded with the resolution of the pneumothorax or consultation with a thoracic surgeon for consideration of VATS bleb resection and mechanical pleurodesis. Each branch node represented the probability of a specific clinical event,^6^^,^^11^, ^12^, ^13^, ^14^, ^15^, ^16^, ^17^, ^18^, ^19^, ^20^, ^21^, ^22^, ^23^, ^24^ and subsequent treatments after initial failure were guided by both expert opinion and the protocols of included clinical trials. VATS was not considered an initial treatment but was included as a next-step option if a small-bore chest tube to suction failed. The outcome of death from PSP was not included in the model due to its rare occurrence.^35^ Terminal nodes of the model represent outcome values based on quality-adjusted life-years (QALYs) and costs. A discount rate of 3% was applied to calculate discounted QALYs and costs in accordance with standard recommendations.^25^

### 2.7 Statistical Analysis

**Figure 2:**
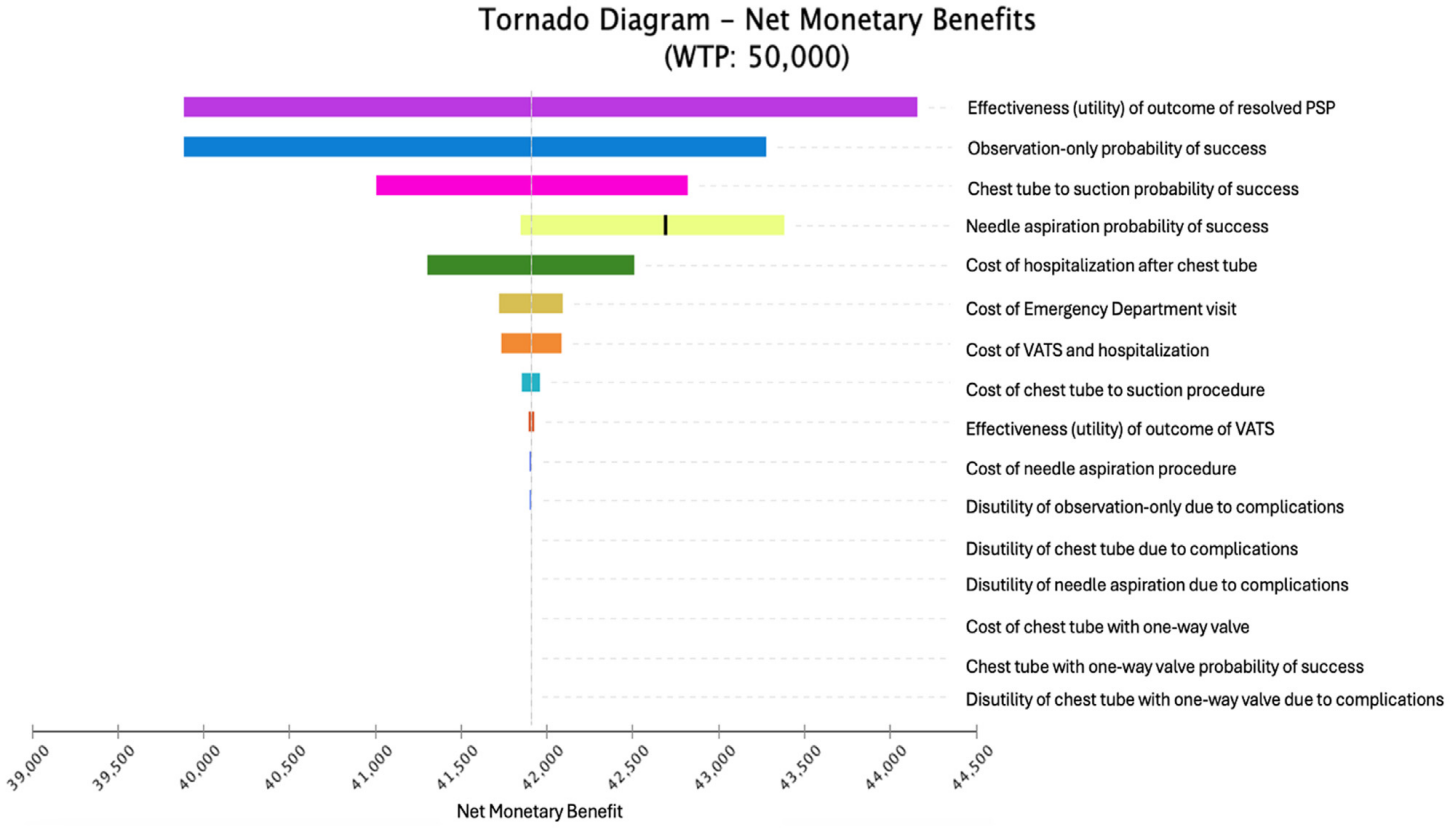
Tornado diagram of 1-way sensitivity analyses of NMB. Tornado diagram illustrating the 1-way sensitivity analyses for net monetary benefit (NMB), highlighting the relative influence of each parameter on the model’s cost-effectiveness results. Longer bars indicate parameters with greater impact on NMB, providing insight into which variables most strongly affect the model’s conclusions.

The 4 treatment strategies were compared using QALYs, total cost of treatment, and NMB. We used a willingness-to-pay (WTP) threshold of $50,000 per QALY.^36^ Cost-effectiveness was assessed from the perspective of the health care organization. Sensitivity analyses were performed to determine how assumptions regarding clinical inputs and cost parameters influenced base case results. One-way sensitivity analyses were represented with a tornado diagram (Fig 2), which demonstrates how our base case estimates for cost-effectiveness would change as a single parameter is varied. A probabilistic sensitivity analysis was conducted by using Monte Carlo simulation to simultaneously assess uncertainty around all key parameters. We performed 5000 simulations with probabilities, outcomes, and costs drawn from the estimated distributions of our model.

## 3 Results

All 4 of the strategies are cost effective as determined by NMB at a WTP threshold of $50,000 per QALY. In the base case analysis, observation only was the most cost-effective strategy. The second most cost-effective strategy was a small-bore chest tube with a 1-way valve followed by needle aspiration and then a small-bore chest tube to suction (Table 4). Observation only has an additional $30,865 NMB compared with a small-bore chest tube to suction.

**Table 4:** Base case cost-effectiveness analysis.

Management strategy	Cost, US $	Incremental cost, US $	Effectiveness, QALY	Incremental effectiveness	NMB (WTP 50,000), US $	△ NMB (WTP 50,000), US $
Observation only	5332		49.82		41,910	
Needle Aspiration	16,766	13,250	48.76	−0.02	29,465	−12445
Small-bore chest tube with 1-way valve	13,086	7754	49.29	−0.01	33,520	−8390
Small-bore chest tube to suction	33,728	28,395	48.76	−0.02	12,831	−29,079

NMB, net monetary benefit; QALY, quality-adjusted life-years; WTP, willingness-to-pay threshold.

**Figure 3:**
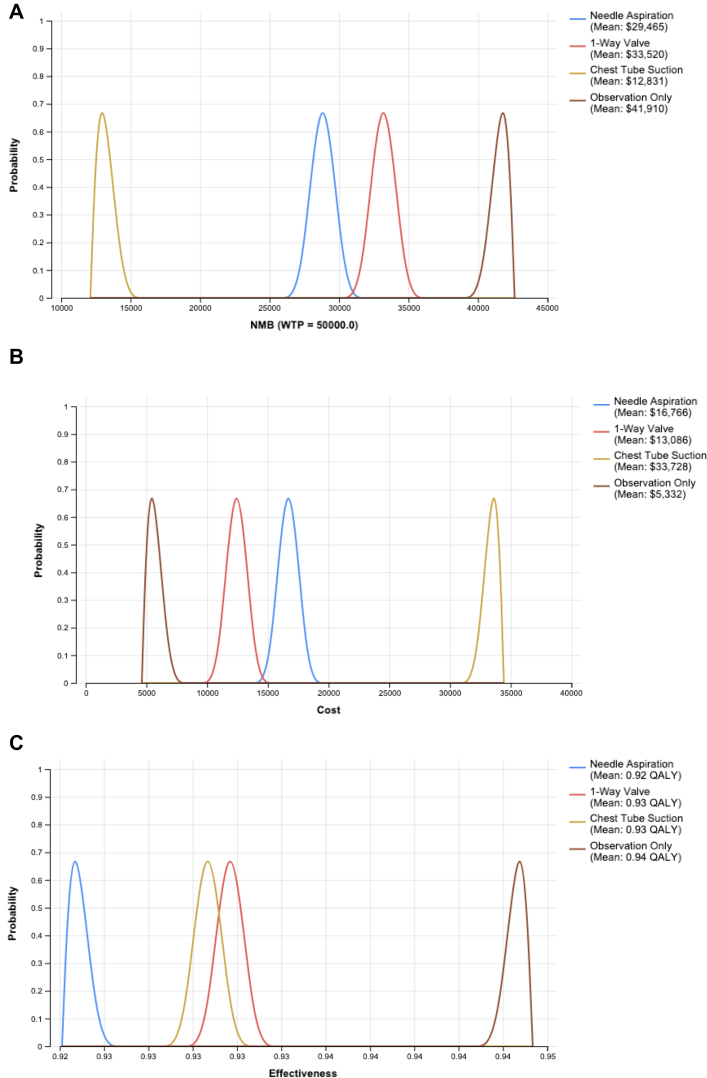
Probability distributions for primary spontaneous pneumothorax treatment strategies. A, Net monetary benefit. B, Cost. C, Effectiveness. Probabilistic sensitivity analysis results showing the distributions of net monetary benefit, total cost, and effectiveness (measured in QALYs) for the 4 treatment strategies. Each curve represents the range of outcomes from 5000 Monte Carlo simulations, illustrating the uncertainty around model inputs and outcomes, and confirming the dominance of the observation only strategy.

One-way sensitivity analyses were performed, which found the most significant contributors to variation in outcomes from our model were the disutility after resolved PSP, the probability of initial success of needle aspiration, and the probability of initial success of observation only (Fig 2). Plots of Monte Carlo simulations for cost, effectiveness, and NMB are shown in Figure 3. Monte Carlo simulations demonstrate small overlap between 1-way valve and needle aspiration for NMB. Observation only was the dominant strategy in all simulations and a small-bore chest tube to suction was always the least cost effective. The majority of simulations found small-bore chest tubes with 1-way valves to be cost-effective compared with needle aspiration (Fig 3).

## 4 Limitations

This analysis has several limitations that should be considered when interpreting the findings. First, although clinical inputs were derived from published RCTs, only 1 trial evaluated the observation only strategy, limiting the robustness and generalizability of conclusions. Moreover, that trial lacked high confidence in demonstrating noninferiority and increased procedures, ED visits, surgeries, or imaging—could significantly drive up costs. These uncertainties highlight the need for further validation of the observation only strategy’s cost-effectiveness in real-world settings. We defined success in the observation only strategy as discharge without intervention, even if the pneumothorax had not fully resolved. Although this may complicate comparisons with strategies that define success as complete resolution, follow-up data showed resolution in these patients at 8 weeks. Aside from the minor disutility associated with a longer duration of pneumothorax, the final outcome was a resolved PSP. This definition results in a higher initial success rate for the observation only strategy—not due to iatrogenic harm from invasive strategies, but due to differing outcome definitions (safe discharge vs immediate lung re-expansion). Much of the cost and clinical data for conservative management approaches (eg, needle aspiration, 1-way valve) come from studies conducted outside the United States, where health care practices, costs, and patient preferences may differ or limit applicability to US health care settings. A further limitation is that some QALY estimates were based on utility values for similar conditions based on the lack of primary data. There are no published disutility studies on VATS for bleb resection and mechanical pleurodesis. The disutility of a long-term complication of VATS was estimated using a published long-term disutility value for moderate asthma (0.83), chosen as a representative of morbidity associated with intermittent dyspnea; this value is comparable with the disutility associated with a similar invasive lung procedure, bronchoscopic therapy for atelectasis (0.8).^34^^,^^37^

This analysis is a study of initial management strategies, and as such, it does not include variation in follow-up costs, which may vary, depending on the health care setting and type. Additionally, we did not include the cost of complications in the analysis. Instead, we used the risk of complications from invasive procedures (eg, chest tube and VATS) to estimate disutility. Given the low incidence of costly complications,^38^ this omission is unlikely to significantly affect the relative cost-effectiveness.

Although increasingly, a WTP threshold of $100,000 per QALY is used,^39^ all treatments in this study are cost effective at the more conservative threshold of $50,000 per QALY, making it unlikely that a higher threshold would influence the general interpretation of our findings.

## 5 Discussion

Based on the included studies, all 4 strategies were found to be cost-effective relative to the WTP threshold. Among them, observation only emerged as the most cost effective, demonstrating the highest NMB, greatest effectiveness, and lowest cost. Although conservative treatment strategies for PSP have been studied for decades,^40^ the standard approach in the United States remains a small-bore chest tube with suction.^41^^,^^42^ By incorporating the cost-effectiveness analysis into the existing efficacy data, our goal was to provide clinicians with additional insight when selecting a management strategy. These findings are provoking and must be considered in the context of US health care practices. Because observation of large PSPs does not represent current clinical practice, it is challenging to compare with other methods due to heterogeneous definitions in the timeframe of resolution. Front-line clinicians or patients may not feel comfortable “doing nothing” and may be subject to increased medicolegal liability in cases of large but hemodynamically stable PSP, which we were unable to model.

We chose to report NMB rather than incremental cost-effectiveness ratio due to its interpretability, particularly when comparing multiple strategies.^43^ NMB assigns a dollar value to the health benefits of a treatment based on a WTP threshold per QALY gained and subtracts its cost.

Given that observation only is not widely practiced in the United States, our findings comparing the invasive strategies remain highly relevant. Among these, small-bore chest tubes with a 1-way valve were the most cost effective, driven by a high initial success rate and the potential for outpatient management. Needle aspiration, with a lower success rate (65.7%), often required repeat procedures. Although complication rates were similar across all strategies, cost differences were substantial—primarily due to hospitalization expenses.

Observation only is a much newer strategy, with only 1 RCT^6^ included in our analysis, compared with multiple RCTs over many years for the other strategies. In this study, 84.6% (137/162) of patients did not require additional ED intervention and were discharged, which is a higher rate than the success of any of the other interventions. It may be difficult for the reader to accept the findings that observation only results in the highest effectiveness. The results of this trial are challenging to compare with the results from studies of the other interventions because by design, the pneumothorax does not resolve at the time of the index visit. Our choice to define success in the model for the observation only group as discharged without intervention is supported by the finding that 94.4% of discharged patients in this study had resolved pneumothorax at their time of follow-up at 8 weeks (118/125), which is similar to the recurrence rate after invasive management.^6^ We included this strategy, given that it is a novel intervention with promising results in the randomized control trial that studied it. However, we believe that many US clinicians or patients may prefer an intervention that resolves the pneumothorax at the time of an ED visit, especially for a large or symptomatic PSP. This may be especially true in populations where follow-up to determine resolution is challenging.

For the clinician who elects to perform an invasive management strategy to resolve the large PSP, our results suggest a small-bore chest tube with a 1-way valve was the most cost-effective strategy, with a high rate of initial success (76.9%), allowing for ambulatory management. However, all of the included studies of small-bore chest tubes with 1-way valves included in this analysis were performed in Europe or Asia; therefore, this may be a less desirable option in a heterogeneous health care system like the United States. Additionally, this strategy does require the patient to leave the ED with the device in place, which may be undesirable for patients.

More conservative strategies like needle aspiration or observation only may be particularly suitable for emerging scenarios where in-person follow-up is not desired from the perspective of the patient or the health system now that postpandemic telemedicine practices have expanded, or where the ED initiating chest tube placement cannot directly ensure follow-up for removal at a subsequent clinic visit.^44^ Figure 3 highlights that needle aspiration may also be performed, as it is cost effective, has some degree of overlap in cost-effectiveness with small-bore chest tube with 1-way valve, and is guideline-concordant with European practice.^45^

This cost-effectiveness analysis indicates that all 4 strategies for managing large PSP are cost effective at a $50,000/QALY threshold. Among them, observation alone was most cost effective, followed by a small-bore chest tube with a 1-way valve, needle aspiration, and finally small-bore chest tube with continuous suction. The observation only approach merits further investigation and may offer an opportunity to align clinical practice with patient preferences and resource stewardship.

## Author Contributions

MLD and MKH conceptualized study and performed literature review.

JH and MLD performed statistical analysis.

RAT, RK, and FF contributed to model design and analysis interpretation.

## Funding and Support

This study was funded by the 10.13039/100007812University of Washington Department of Emergency Medicine Critical Care Resident/Fellow Research Grant.

## Conflict of Interest

Andrew Taylor receives unrelated support from grants from the National Institutes of Health (NIH), Gordon and Betty Moore Foundation, Federal Drug Administration, the Agency for Healthcare Research & Quality (AHRQ), and Beckman Coulter, Inc. as well as options from Vera Health for serving as an advisor. Farhood Farjah receives unrelated support from grants from the NIH. M. Kennedy Hall has received unrelated support from Sonomotion, a commercial ultrasound system.
