# Supplementary material for: Management of Primary Spontaneous Pneumothorax in the Emergency Department: A Cost-Effectiveness Analysis
Source: J Am Coll Emerg Physicians Open. 2025 Jul 2;6(4):100209. doi: 10.1016/j.acepjo.2025.100209 (PMC12271783; doi:10.1016/j.acepjo.2025.100209)
Supplement: Table S1 [file mmc1.docx]

Supplemental Table 1.

Included studies for Model Estimates

| Study | Sample size | Country | Brief Description |
| --- | --- | --- | --- |
| Ayed 2006^11^ | 137 | Kuwait | Comparison of needle aspiration versus tube drainage |
| Ho 2011^12^ | 48 | Singapore | Comparison of minichest tube and needle aspiration in outpatient management |
| Noppen 2002^13^ | 60 | Belgium | Multicenter study of needle aspiration versus chest tube drainage |
| Thelle 2017^14^ | 79 | Norway | Comparison of needle aspiration and chest tube drainage |
| Kim 2019^15^ | 40 | Korea | Comparison of needle aspiration to closed thoracostomy |
| Ramouz 2018^16^ | 70 | Iran | Comparison of chest tube drainage versus needle aspiration |
| Korczyński 2015^23^ | 49 | Poland | Comparison of small bore catheter aspiration and chest tube drainage |
| Parlak 2012^17^ | 56 | Netherlands | Comparison of needle aspiration versus conventional chest tube drainage |
| Andrivet 1995^18^ | 61 | France | Comparison of thoracic drainage versus immediate or delayed needle aspiration |
| Ma 2007^19^ | 46 | China | Pleural drainage using central venous catheter and simple aspiration versus aspiration with thoracentesis |
| Oh 2003^20^ | 57 | Korea | Comparison of simple manual aspiration and chest tube drainage |
| Hallifax 2020^21^ | 236 | UK | Multicenter comparison of chest tube with one-way valve versus needle aspiration and/or chest tube to suction |
| Röggla 1996^24^ | 30 | Austria | Comparison of thoracic vent versus conventional intercostal tube drainage |
| Harvey 1994^22^ | 73 | UK | Comparison of simple aspiration versus intercostal tube drainage |
| Brown 2020^6^ | 162 | Australia/New Zealand | Multicenter comparison of observation only versus chest tube with one-way valve or needle aspiration |
